# Effects of Baccharin Isolated from Brazilian Green Propolis on Adipocyte Differentiation and Hyperglycemia in *ob/ob* Diabetic Mice

**DOI:** 10.3390/ijms22136954

**Published:** 2021-06-28

**Authors:** Akio Watanabe, Marília Oliveira de Almeida, Yusuke Deguchi, Ryuzo Kozuka, Caroline Arruda, Andresa Aparecida Berretta, Jairo Kenupp Bastos, Je-Tae Woo, Takayuki Yonezawa

**Affiliations:** 1 Research Institute for Biological Functions, Chubu University, Kasugai, Aichi 487-8501, Japan; akio-wa@jumonji-u.ac.jp (A.W.); jwoo@isc.chubu.ac.jp (J.-T.W.); 2 Department of Food Science, Faculty of Human Life, Jumonji University, Niiza, Saitama 352-8510, Japan; 3 School of Pharmaceutical Sciences of Ribeirão Preto, University of São Paulo, Avenida do Café, s/n, Ribeirão Preto 14040-903, SP, Brazil; marilia_almeida11@yahoo.com.br (M.O.d.A.); carolinearruda1501@gmail.com (C.A.); jkbastos@fcfrp.usp.br (J.K.B.); 4 Department of Biological Chemistry, Chubu University, Kasugai, Aichi 487-8501, Japan; deguc3@gmail.com (Y.D.); hachiya88neko@gmail.com (R.K.); 5 Laboratory of Research, Development and Innovation, Apis Flora Indl. Coml. Ltda., Ribeirão Preto 14020-670, SP, Brazil; andresa.berretta@apisflora.com.br

**Keywords:** diabetes, propolis, baccharin, PPARγ

## Abstract

Propolis is a honeybee product with various biological activities, including antidiabetic effects. We previously reported that artepillin C, a prenylated cinnamic acid derivative isolated from Brazilian green propolis, acts as a peroxisome proliferator-activated receptor γ (PPARγ) ligand and promotes adipocyte differentiation. In this study, we examined the effect of baccharin, another major component of Brazilian green propolis, on adipocyte differentiation. The treatment of mouse 3T3-L1 preadipocytes with baccharin resulted in increased lipid accumulation, cellular triglyceride levels, glycerol-3-phosphate dehydrogenase activity, and glucose uptake. The mRNA expression levels of PPARγ and its target genes were also increased by baccharin treatment. Furthermore, baccharin enhanced PPARγ-dependent luciferase activity, suggesting that baccharin promotes adipocyte differentiation via PPARγ activation. In diabetic *ob/ob* mice, intraperitoneal administration of 50 mg/kg baccharin significantly improved blood glucose levels. Our results suggest that baccharin has a hypoglycemic effect on glucose metabolic disorders, such as type 2 diabetes mellitus.

## 1. Introduction

Propolis is a sticky, resinous material that honeybees (*Apis mellifera* L.) collect from plants and mix with wax and other secretions to maintain hive temperatures and seal cracks in the hive [1]. In many countries, propolis has been used since ancient times in folk medicine, health drinks, and foods because of its antioxidant, antimicrobial, anti-inflammatory, antiobesity, antidiabetes, and antiulcer properties [2,3,4,5,6]. Propolis has a particularly complex composition that includes beeswax, resin, and volatile compounds as main constituents [7]. The biological activity of propolis is attributed to plant-derived substances. *Baccharis dracunculifolia* is the main botanical source of Brazilian green propolis, which characteristically contains cinnamic acid derivatives, such as p-coumaric acid, baccharin, drupanin, and artepillin C [8,9].

The worldwide prevalence of type 2 diabetes is increasing annually [10,11]. Type 2 diabetes is a metabolic disorder characterized by resistance to insulin, a unique hormone that controls glucose absorption to maintain whole-body glucose homeostasis [12,13]. Defects in insulin sensitivity in adipose tissue, liver tissue, and muscle disrupt blood glucose homeostasis, resulting in hyperglycemia [14]. Due to the increased rates of type 2 diabetes, effective agents that improve insulin sensitivity are urgently needed. Hypertrophic adipocytes secrete inflammatory cytokines, such as tumor necrosis factor-α (TNF-α) and monocyte chemoattractant protein (MCP-1), leading to insulin resistance [15]. In contrast, adiponectin secreted by adipocytes improves insulin resistance, enhances muscle glucose uptake, stimulates liver fatty acid oxidation, and suppresses hepatic glucose production [16].

Peroxisome proliferator-activated receptor γ (PPARγ), a member of the nuclear receptor superfamily, is a key factor in adipogenesis. Thiazolidinediones, such as rosiglitazone and pioglitazone, are PPARγ agonists reported to improve insulin sensitivity by inducing adipocyte differentiation and increasing adiponectin production in diabetic patients. However, thiazolidinediones are associated with weight gain and other side effects [17,18,19]. Therefore, identifying compounds that improve insulin resistance without increasing body weight is needed to develop effective antidiabetic agents [20].

We previously reported that artepillin C, a component of Brazilian green propolis, promotes adipocyte differentiation and glucose uptake in part via PPARγ activation [4]. Others reported that adipocyte differentiation is also enhanced by drupanin isolated from Brazilian green propolis [21]. In this study, we examined the effects of baccharin, another prenylated cinnamic acid derivative present in Brazilian green propolis, on adipocyte differentiation in vitro and glucose metabolism in vivo using obese diabetic *ob/ob* mice.

## 2. Results

### 2.1. Effects of Baccharin on 3T3-L1 Adipocyte Differentiation

**Figure 1:**
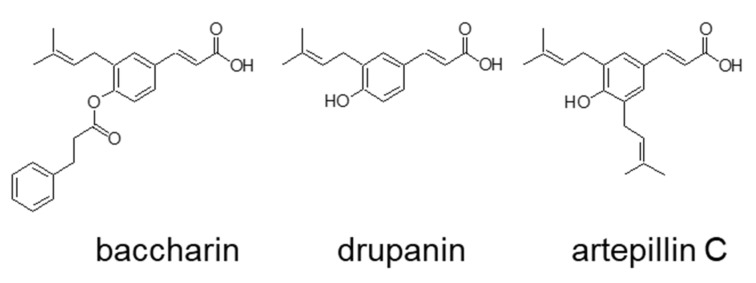
Chemical structure of baccharin, drupanin, and artepillin C.

To investigate the effects of prenylated cinnamic acid derivatives isolated from Brazilian green propolis on adipocyte differentiation, 3T3-L1 preadipocytes were treated with baccharin, drupanin, or artepillin C (Figure 1) in the presence of insulin.

**Figure 2:**
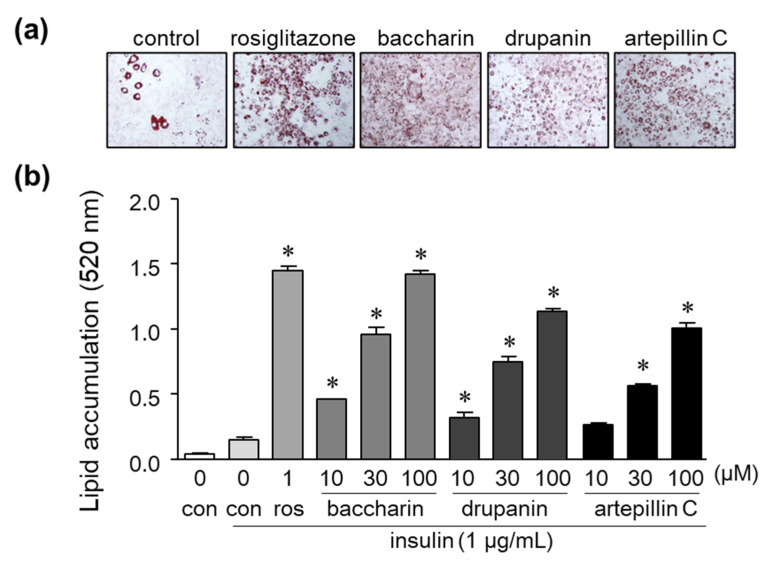
Effect of baccharin, drupanin, and artepillin C on lipid accumulation in 3T3-L1 cells. (**a**) 3T3-L1 preadipocytes were incubated in the presence of insulin (1 µg/mL) with rosiglitazone (1 µM), baccharin (30 µM), drupanin (30 µM), or artepillin C (30 µM) as described in the Methods section of this paper. The cells were then stained with Oil Red O and photographed. (**b**) The accumulated lipids stained with Oil Red O were dissolved, and the absorbance was measured using a spectrometer. Values are expressed as means ± SEM (*n* = 3). * *p* < 0.05 compared with the control in the presence of insulin. ros, rosiglitazone; con, control.

The treatment of cells with artepillin C and drupanin increased the amount of lipid droplets stained by Oil Red O to a similar degree as the PPARγ ligand rosiglitazone, which served as a positive control (Figure 2a). Baccharin treatment also significantly and dose-dependently increased the amount of accumulated lipids, as reflected by the increased numbers of lipid droplets positive for Oil Red O staining (Figure 2a). Moreover, the amount of lipid droplets in cells treated with baccharin was the highest among the three cinnamic acid derivatives from green propolis (Figure 2a,b).

**Figure 3:**
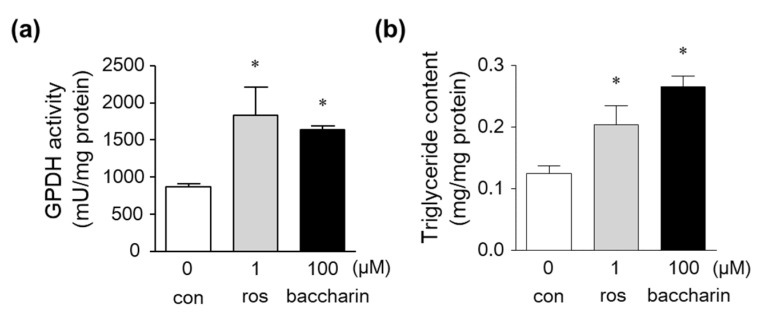
Effect of baccharin on GPDH activity and triglyceride levels in 3T3-L1 cells. Then, 3T3-L1 preadipocytes were cultured in the presence of insulin (1 µg/mL) with rosiglitazone (1 µM) or baccharin (100 µM). (**a**) GPDH activity and (**b**) triglyceride levels in the cultured cells were measured. Values are expressed as means ± SEM (*n* = 3). * *p* < 0.05 compared with the control in the presence of insulin. ros, rosiglitazone; con, control.

Glycerol-3-phosphate dehydrogenase (GPDH) is a marker of adipocytes and regulates the conversion of glycerol into triglyceride [22]. Intracellular GPDH activity and triglyceride levels in 3T3-L1 preadipocytes treated with the PPARγ ligand rosiglitazone were significantly increased (Figure 3a,b). Baccharin treatment also significantly increased GPDH activity and triglyceride levels (Figure 3a,b), indicating that baccharin induces the differentiation of preadipocytes into adipocytes.

**Figure 4:**
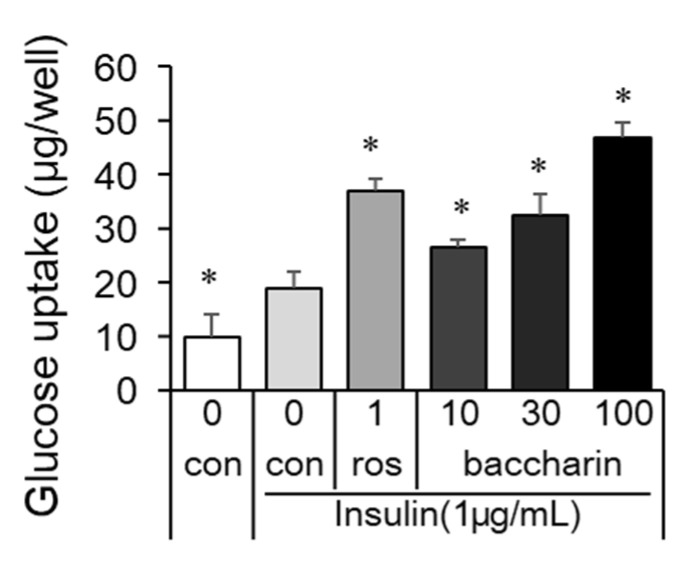
Effect of baccharin on glucose uptake in 3T3-L1 cells. In the presence of insulin (1 µg/mL), 3T3-L1 preadipocytes were cultured with rosiglitazone (1 µM) or baccharin (10–100 µM). The glucose concentrations in cultured medium were measured, and the differences before culture and after culture were calculated as glucose uptake. Values are expressed as means ± SEM (*n* = 3). * *p* < 0.05 compared with the control in the presence of insulin. ros, rosiglitazone; con, control.

Additionally, a glucose uptake assay was performed after cell culture with baccharin or rosiglitazone in the presence of insulin. The positive control rosiglitazone (1 µM) enhanced glucose uptake (Figure 4). Similarly, baccharin dose-dependently increased the amount of glucose uptake. Thus, these results indicated that baccharin enhanced adipocyte differentiation and glucose uptake.

### 2.2. Effects of Baccharin on the mRNA Expression of Adipocyte-Related Genes and PPARγ-Dependent Transcriptional Activity

**Figure 5:**
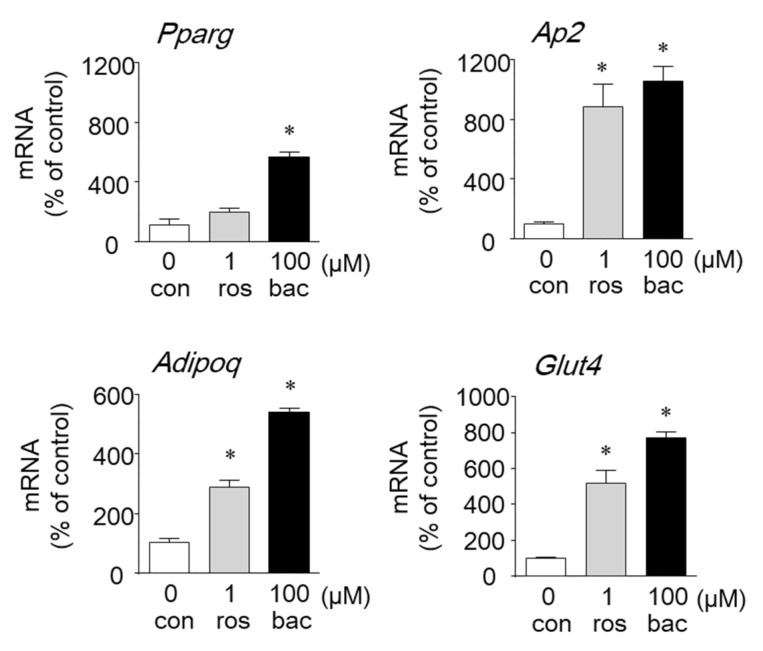
Effect of baccharin on the mRNA expression of genes related to adipocyte differentiation. In the presence of insulin (1 µg/mL), 3T3-L1 preadipocytes were cultured with rosiglitazone (ros: 1 µM) or baccharin (bac: 100 µM). Total RNA was isolated, and cDNA was synthesized. The mRNA expression of each gene was determined by real-time RT-PCR. Values are expressed as means ± SEM (*n* = 3). * *p* < 0.05 compared with the control (con) in the presence of insulin.

PPARγ is a key transcriptional factor in adipogenesis and regulates the expression of adipocyte-specific genes. To examine whether baccharin affects the expression of genes involved in adipogenesis, we evaluated changes in gene expression in 3T3-L1 cells treated with baccharin by real-time RT-PCR. The mRNA expression level of PPARγ was significantly increased by baccharin (100 μM) (Figure 5).

Baccharin also increased the mRNA expression of the PPARγ-target genes *Ap2*, *Glut4*, and Adipoq (adiponectin gene) compared with 1 µM rosiglitazone (Figure 5).

**Figure 6:**
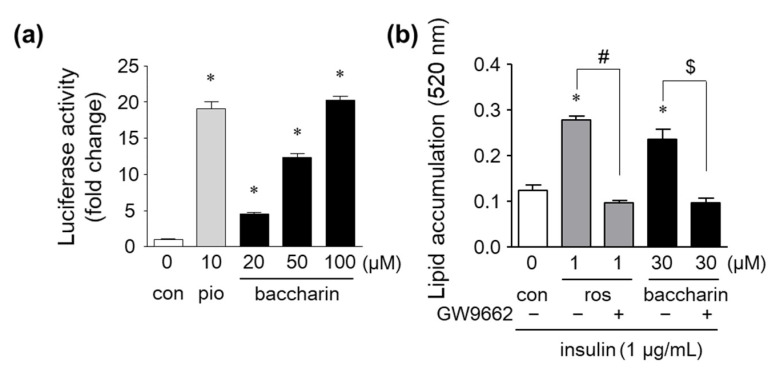
Effects of baccharin on PPARγ-dependent transcriptional activity and PPARγ antagonism on baccharin-induced lipid accumulation. (**a**) CV-1 cells were transfected with reporter genes and cultured with pioglitazone or baccharin for 48 h. PPARγ-dependent transcriptional activity was measured by a dual-luciferase reporter assay. (**b**) 3T3-L1 cells were cultured in the presence of rosiglitazone (1 µM) or baccharin (30 µM) with or without GW9662 (10 µM) and then stained with Oil Red O. The amount of accumulated lipid droplets stained with Oil Red O was determined. Values are expressed as means ± SEM (*n* = 3). * *p* < 0.05 compared with the control; # *p* < 0.05 versus 1 µM rosiglitazone-treated cells; $ *p* < 0.05 versus 30 µM baccharin-treated cells. pio, pioglitazone. ros, rosiglitazone.

Next, PPARγ-dependent transcriptional activity was determined by a luciferase reporter gene assay. Baccharin treatment markedly and dose-dependently elevated PPARγ-dependent luciferase activity to similar levels as those observed for pioglitazone-treated cells (Figure 6a). Furthermore, baccharin-induced lipid accumulation was suppressed in the presence of GW9662, a PPARγ antagonist (Figure 6b). These results suggest that baccharin activates a PPARγ-dependent pathway, and the action might be associated with the promotion of adipocyte differentiation.

### 2.3. Effects of Baccharin on Glucose Metabolism in Obese Diabetic ob/ob Mice

Using the *ob/ob* mouse model of type 2 diabetes, we next assessed the effects of baccharin on glucose metabolism in vivo. There were no significant differences in body weight, liver weight, or white adipose tissue weight after 5 weeks of treatment with baccharin (50 mg/kg, i.p.) (Table 1). 

In contrast, non-fasting and fasting blood glucose levels were significantly lower in the baccharin-treated group compared with the vehicle-treated group at 5 weeks (Table 1). The plasma adiponectin levels did not differ significantly between the vehicle-treated and baccharin-treated groups. However, insulin levels tended to be reduced in the baccharin-treated group, and the homeostasis model assessment of insulin resistance (HOMA-IR) index was significantly improved (Table 1).

## 3. Discussion

Brazilian green propolis has traditionally been used to treat several health disorders [5,23], and it may provide a good source of antidiabetic factors [5,24]. Baccharin, artepillin C, and drupanin are the main cinnamic acid derivatives found in Brazilian green propolis. We previously reported that artepillin C induced adipocyte differentiation [4], and drupanin was also shown to enhance adipocyte differentiation [22]. However, the effect of baccharin on adipocyte differentiation was unknown. Here, we investigated the effects of three prenylated cinnamic acid derivatives (baccharin, artepillin C, and drupanin) from Brazilian green propolis and found that all three components enhanced adipocyte differentiation.

When 3T3-L1 preadipocytes were treated with baccharin in the presence of insulin, the number of lipid droplets, cellular triglyceride levels, and GPDH activity were increased. These findings indicated that baccharin promoted the differentiation of preadipocytes into adipocytes. Further analyses using real-time RT-PCR showed an increase in the expression of PPARγ and its target genes (*Ap2*, *Glut4*, and *Adipoq*) following baccharin treatment, suggesting that baccharin enhances adipocyte differentiation in part by modulating the expression of genes critical for adipogenesis. We and others reported that artepillin C and drupanin enhanced adipocyte differentiation via the PPARγ signaling pathway [4,21]. Therefore, we examined whether baccharin serves as a PPARγ activator to affect the expression of adipogenesis-related genes. In a reporter gene assay, baccharin enhanced PPARγ-dependent transcriptional activity. In addition, the number of lipid droplets in baccharin-treated cells was significantly decreased following treatment with a PPARγ antagonist. These results suggested that baccharin promotes adipocyte differentiation via PPARγ agonist activity. Baccharin treatment also enhanced GLUT4 mRNA expression and glucose uptake in differentiated 3T3-L1 adipocytes. Therefore, baccharin is expected to have an antidiabetic effect. It has been reported that cinnamic acid and *p*-coumaric acid induced the expression of PPARγ and GLUT4 to promote glucose uptake in 3T3-L1 cells [25], indicating the importance of the structure of cinnamic acid in the action of baccharin. Structure–activity/property relationship of prenylated *p*-coumaric acid derivatives from Brazilian green propolis extract on absorption, metabolism and other actions are under investigation.

Although several reports have demonstrated that Brazilian green propolis extracts have hypoglycemic effects [26,27], there are no reports on the influence of treatment with baccharin alone in vivo. In the present animal study, we investigated the effects of baccharin on glucose metabolism and insulin sensitivity in obese diabetic *ob/ob* mice. The baccharin (50 mg/kg, i.p.)-treated group exhibited significantly reduced blood glucose levels and HOMA-IR values at 5 weeks compared with the vehicle-treated group, indicating that baccharin ameliorated hyperglycemia and insulin resistance.

Treatment with baccharin increased adiponectin gene expression in adipocyte cell cultures. However, there was no difference in the serum protein levels of adiponectin between control *ob/ob* mice and baccharin-administrated *ob/ob* mice. To reveal the contribution of adiponectin to the action of baccharin, it is necessary to verify the effect of baccharin on the expression of adiponectin and other adipokines, such as leptin, resistin, MCP-1, TNF-α, and interleukin-6, in serum and adipose tissues in future research.

It should be noted that baccharin and the PPARγ agonist activated PPARγ-dependent transcription, but baccharin administration did not induce the side effect of weight gain observed in patients treated with thiazolidinediones and PPARγ agonists. Nuclear receptors are known to mobilize different cofactors in a ligand-dependent manner to elicit various actions. For example, it has been reported that telmisartan activates PPARγ through a coactivator different from the full agonist pioglitazone. Moreover, telmisartan does not promote weight gain in high-fat diet model mice, unlike pioglitazone [28,29]. The possibility that baccharin also acts as a partial agonist of PPARγ will be clarified in a future analysis of the direct interaction and binding between ligands and receptors. Together, our results indicate that baccharin provides a unique candidate to ameliorate hyperglycemia and insulin resistance without promoting weight gain.

In conclusion, the present study has shown for the first time that baccharin enhances adipocyte differentiation via PPARγ activation in a cell model and that baccharin administration reduces blood glucose levels in the *ob/ob* mouse model of type 2 diabetes. Our results provide important implications for the use of Brazilian green propolis extracts or baccharin to prevent insulin resistance.

## 4. Materials and Methods

### 4.1. Materials

Brazilian green propolis was provided by Apis Flora Comercial, Ltda., Ribeirão Preto, SP, Brazil. High-performance liquid chromatography (HPLC) grade methanol, acetate, hexane, and ethyl acetate were purchased from Mallinckrodt Co. (Xalostoc, Mexico), and ultra-pure water was obtained after purification with Milli-Q plus filter systems (Millipore, Bedford, MA, USA). For vacuum liquid chromatography, silica gels with over 90% of particles <45 μm in size were purchased from Merck Co. (Darmstadt, Germany). Normal-phase analytical thin layer chromatography (TLC) media was also purchased from Merck (Cat No. 60F254). Insulin, Dulbecco’s modified Eagle’s medium (DMEM), penicillin–streptomycin, calf serum (CS), and fetal bovine serum (FBS) were purchased from Thermo Fisher Scientific (Waltham, MA, USA). The PPARγ antagonist GW9662 was purchased from FUJIFILM Wako (Osaka, Japan). All other reagents were of the highest grade commercially available.

### 4.2. Isolation of Baccharin from Brazilian Green Propolis

Crude ethanolic extract (87 g) from green propolis (200 g) was subjected to vacuum liquid chromatography. Silica gel 60 H was added to a 13.5-cm diameter sintered glass column to a height of 5.5 cm, and hexane was used to efficiently pack the column. The sample was applied to the column, and compounds were eluted using a hexane–acetate gradient of 95% to 70% for the collection of 200 fractions that were analyzed by TLC and HPLC and combined into six subfractions. The subfractions were subjected to preparative reverse-phase HPLC, which yielded 2.5 g, 100 mg, and 3 g of baccharin, drupanin, and artepillin C, respectively. The structures of the isolated compounds (Figure 1) were identified based on the comparison of 1H NMR and 13C NMR spectroscopic data with high-resolution mass spectra (ESI-MS), as previously published [6,30,31].

### 4.3. Cell Culture and Adipocyte Differentiation

Mouse 3T3-L1 preadipocytes were obtained from the RIKEN BRC (Tsukuba, Japan). The cells were cultured in DMEM supplemented with 10% heat-inactivated CS, penicillin (100 units/mL), and streptomycin (100 μg/mL) in an atmosphere of 5% CO_2_ at 37 °C.

For adipocyte differentiation, 3T3-L1 preadipocytes (5 × 10^3^ cells/well) were seeded in 96-well cell-culture plates and cultured until confluence in 10% CS-DMEM. Then, the cells were treated with or without each of the test samples in the presence of insulin (1 μg/mL) in 10% FBS-DMEM for 9 days. On days 3 and 6, the medium was exchanged with fresh media.

### 4.4. Oil Red O Staining

After culture, the cells were fixed with 10% formalin for 1 h at room temperature and stained with filtered 0.2% Oil Red O in 60% isopropanol for 1 h. After staining, the wells were washed with 60% isopropanol and distilled water. Then, the cells were photographed under a microscope. Stained lipid droplets were extracted using isopropanol, and the absorbance at 520 nm was measured spectrophotometrically as an index of lipid content.

### 4.5. Triglyceride Levels and GPDH Activity

To measure triglyceride levels and GPDH activity, 3T3-L1 preadipocytes (5 × 10^4^ cells/well) were seeded in 24-well cell culture plates and incubated in 10% CS-DMEM. After reaching confluence, the cells were cultured with insulin (1 μg/mL) in the presence of baccharin in 10% FBS-DMEM for 9 days. The cells were washed twice with ice-cold PBS, harvested in ice-cold 25 mM Tris-HCl (pH 7.5) buffer containing 1 mM ethylenediaminetetraacetic acid, and lysed by brief sonication. The triglyceride levels of the lysate were quantified using a Triglyceride E-test kit (Wako) and expressed as mg/mg protein. GPDH activity was measured using a GPDH Activity Assay Kit (Takara Bio, Shiga, Japan) and expressed as mU/mg protein. Protein concentration was determined using a bicinchoninic acid (BCA) Protein Assay Kit (Thermo Fisher Scientific, Tokyo, Japan).

### 4.6. Glucose Uptake Assay

After 3T3-L1 cell culture in 96-well plates under adipocyte differentiation conditions, the glucose concentrations in the cultured medium were measured using the glucose CII test kit (FUJIFILM Wako) with a microplate reader (TECAN Infinite F50, absorbance at 505 nm). The amounts of glucose uptake were calculated as the differences in glucose concentrations compared to the uncultured medium.

### 4.7. RNA Preparation and qRT-PCR

Adipocyte differentiation was performed in 24-well cell-culture plates as described above. Total RNA was extracted using TRIzol reagent (Life Technologies, Carlsbad, CA, USA) and an RNeasy Mini Kit (Qiagen, Venlo, Netherlands). cDNA was synthesized using random primers and PrimeScript Reverse Transcriptase (Takara Bio, Shiga, Japan). The target cDNAs were amplified using SYBR Fast qPCR Mix (Takara Bio) and gene-specific primers. The primer sequences used for the PCR amplifications were: *Adipoq*, 5’-GTTGCAAGCTCTCCTGTTCC-3’ and 5’-CTTGCCAGTGCTGTTGTCAT-3’; *aP2*, 5’-CAACCTGTGTGATGCCTTTGTG-3’ and 5’-CTCTTCCTTTGGCTCATGCC-3’; *Glut4*, 5’- CCCCGATACCTCTACATCATC-3’ and 5’-GCATCAGACACATCAGCCCAG-3’; *β-actin*, 5’-TGTTACCAACTGGGACGACA-3’ and 5’-CTCTCAGCTGTGGTGGTGAA-3’; and PPARγ, 5’-GCTGTTATGGGTGAAACTCTG-3’ and 5’-ATAAGGTGGAGATGCAGGTTC-3’. PCR conditions were as follows: initial denaturation at 95 °C for 60 s followed by 40 cycles of 95 °C for 15 s and 60 °C for 60 s. PCR products were measured using a Light Cycler 96 instrument (Roche Diagnostics, Basel, Switzerland). Data are expressed as fold-differences normalized to β-actin levels.

### 4.8. Reporter Gene Assay for PPARγ

The reporter gene assay for PPARγ was performed by the Northern Advancement Center for Science & Technology (NOASTEC, Sapporo, Japan). PPARγ agonist activity was evaluated using a luciferase reporter gene assay. CV-1 cells derived from an African green monkey kidney (Cell Resource Center for Biomedical Research, Institute of Development, Aging and Cancer, Tohoku University, Miyagi, Japan) were seeded at 2 × 10^5^ cells/well in 6-well plates and cultured overnight in 10% FBS-DMEM. The cells were then transiently transfected with 1 μg of the expression plasmid pGAL4-PPARγ and 0.9 μg of pGal4-Luc together with 0.1 μg of pGL4.75 hRluc-CMV (Promega, Madison, WI, USA) per well using X-tremeGENE HP DNA Transfection Reagent (Roche Diagnostics, GmbH, Mannheim, Germany). After 6 h incubation, the cells were harvested and then plated again at 1.6 × 10^4^ cells/well in 96-well plates with 10% FBS-DMEM containing 20, 50, or 100 µM baccharin. The PPARγ agonist pioglitazone (10 μM) and DMSO (0.5%) were used as a positive and negative control, respectively. After a 48 h incubation, the cells were washed with PBS, lysed, and the activity of firefly and *Renilla* luciferase was measured using a dual luciferase reporter assay system (Promega) with a luminometer (Luminescencer, AB-2350EX, ATTO, Tokyo, Japan). Luciferase activity was corrected for transfection efficiency based on the activity of the internal control *Renilla* luciferase.

### 4.9. Treatment of Obese Type 2 Diabetic ob/ob Mice

C57BL/6J (normal) and C57BL/6J-*ob/ob* (*ob/ob*) mice were purchased from SLC Japan (Shizuoka, Japan) at 5 weeks of age. The mice were individually housed under temperature- (22 °C ± 2 °C) and humidity-controlled conditions with a 12 h light/dark cycle and given free access to water and food. The mice were allowed to adapt to these conditions for 1 week before the beginning of the experimental protocol. The *ob/ob* mice were divided into 3 groups (*n* = 9–10) based on body weight and blood glucose levels. The normal and ob/ob control mice were administered the vehicle alone (1% Tween-80 and 5% DMSO) intraperitoneally. Baccharin (10 or 50 mg/kg body weight) was administered to *ob/ob* mice by intraperitoneal (i.p.) injection once daily for 5 weeks. During the experimental period, the mice had free access to water and food, and their body weights were measured daily. Their fasting and non-fasting blood glucose levels were measured weekly using a blood glucose monitoring system (Accu-Chek Aviva, Roche) to sample blood from the tail vein of the mice. At the end of the 5-week treatment period, the mice were sacrificed by cervical dislocation, and blood was collected. Plasma samples were obtained by centrifugation at 1200 g for 15 min at 4 °C using heparin. The separated plasma samples were stored at −80 °C until analysis. The liver and whole white adipose tissue that included all mesenteric, perinephric, testicular, and subcutaneous tissues were excised immediately, rinsed, weighed, and frozen on dry ice for storage at −80 °C. Plasma insulin and adiponectin levels were measured by enzyme-linked immunosorbent assay (ELISA) (Mouse Insulin ELISA Kit, Shibayagi, Ibaragi, Japan and Mouse Adiponectin ELISA Kit, Otsuka Pharmaceutical Corporation, Osaka, Japan) in accordance with the manufacturers’ protocols. The HOMA-IR was calculated from the fasting blood glucose and insulin levels using the following formula: fasting blood glucose level (mg/dL) × fasting insulin level (ng/mL)/405.

### 4.10. Statistical Analysis

Statistical analysis was performed using GraphPad Prism 5.0 software (GraphPad Software, San Diego, CA, USA). The data are expressed as means ± SEM. One-way ANOVA and Dunnett’s test were performed. Differences were considered significant at *p* < 0.05.

## Figures and Tables

**Table 1:** Effects of baccharin on the body and organ weights and plasma biochemical parameters in normal and *ob/ob* mice.

	Normal Control	*ob/ob* Control	*ob/ob* Baccharin 10 mg/kg	*ob/ob* Baccharin 50 mg/kg
Body weight (g)	22.1 ± 0.4 *	39.7 ± 1.2	39.4 ± 1.8	37.7 ± 1.1
Liver weight (g/100 g body weight)	4.39 ± 0.09 *	6.30 ± 0.36	6.21 ± 0.34	5.64 ± 0.23
White adipose tissue weight (g/100 g body weight)	2.52 ± 0.17 *	18.95 ± 0.45	17.65 ± 0.65	17.13 ± 0.47
Fasting blood glucose (mg/dL)	92.8 ± 3.4 *	308 ± 40	340 ± 33	180 ± 35 *
Non-fasting blood glucose (mg/dL)	163.4 ± 7.3 *	362.0 ± 29.4	364.4 ± 32.8	239.3 ± 43.3 *
Plasma insulin (ng/mL)	N.D.	4.04 ± 0.64	4.94 ± 0.83	1.98 ± 0.72
Plasma adiponectin (µg/mL)	13.6 ± 0.9	20.3 ± 3.6	20.8 ± 3.1	17.9 ± 2.6
HOMA-IR	N.D.	75.4 ± 10.1	107.0 ± 20.5	20.0 ± 6.2 *

Values are expressed as means ± SEM (*n* = 9–10). * *p* < 0.05 compared with the *ob/ob* control. N.D., not detected.

## References

[1] De Oliveira P., Lima I.D.S., Munari C., Bastos J., Filho A.D.S., Tavares D. (2014). Comparative Evaluation of Antiproliferative Effects of Brazilian Green Propolis, Its Main Source Baccharis dracunculifolia, and Their Major Constituents Artepillin C and Baccharin. Planta Med..

[2] Berretta A.A., Arruda C., Miguel F.G., Baptista N., Nascimento A.P., Oliveira F.M., Hori J.I., Barud H.D.S., Damaso C.R.B., Ramos C. (2017). Functional Properties of Brazilian Propolis: From Chemical Composition Until the Market. Superfood Funct. Food Overv. Process. Util..

[3] Ikeda R., Yanagisawa M., Takahashi N., Kawada T., Kumazawa S., Yamaotsu N., Nakagome I., Hirono S., Tsuda T. (2011). Brazilian propolis-derived components inhibit TNF-α-mediated downregulation of adiponectin expression via different mechanisms in 3T3-L1 adipocytes. Biochim. Biophys. Acta (BBA)-Gen. Subj..

[4] Choi S.S., Cha B.Y., Iida K., Lee Y.S., Yonezawa T., Teruya T., Nagai T., Woo J.T. (2011). Artepillin C, as a PPARγ ligand, en-hances adipocyte differentiation and glucose uptake in 3T3-L1 cells. Biochem. Pharmacol..

[5] Kitamura H., Naoe Y., Kimura S., Miyamoto T., Okamoto S., Toda C., Shimamoto S., Iwanaga T., Miyoshi I. (2013). Beneficial effects of Brazilian propolis on type 2 diabetes in ob/ob mice: Possible involvement of immune cells in mesenteric adipose tis-sue. Adipocyte.

[6] Costa P., Almeida M.O., Lemos M., Arruda C., Casoti R., Somensi L.B., Boeing T., Mariott M., da Silva R.D.C.M.V.D.A.F., Stein B.D.P. (2018). Artepillin C, drupanin, aromadendrin-4′-O-methyl-ether and kaempferide from Brazilian green propolis promote gastroprotective action by diversified mode of action. J. Ethnopharmacol..

[7] Viviane C.T., Helia H.S., Glaucia M.P., Yong K.P. (2013). Recent progress of propolis for its biological and chemical composi-tions and its botanical origin. Evid. Based Complement Alternat. Med..

[8] Búfalo M.C., Candeias J.M., Sousa J.P., Bastos J.K., Sforcin J.M. (2010). In vitro cytotoxic activity of Baccharis dracunculifolia and propolis against HEp-2 cells. Nat. Prod. Res..

[9] Salatino A., Teixeira É.W., Negri G., Message D. (2005). Origin and Chemical Variation of Brazilian Propolis. Evid. -Based Complement. Altern. Med..

[10] Seidell J. (2000). Obesity, insulin resistance and diabetes—A worldwide epidemic. Br. J. Nutr..

[11] Lam D.W., Le Roith D. (2012). The worldwide diabetes epidemic. Curr. Opin. Endocrinol. Diabetes Obes..

[12] Reaven G.M. (1988). Role of insulin resistance in human disease. Diabetes.

[13] Kahn B.B. (1998). Type 2 Diabetes: When Insulin Secretion Fails to Compensate for Insulin Resistance. Cell.

[14] Kahn B.B., Flier J.S. (2000). Obesity and insulin resistance. J. Clin. Investig..

[15] Daniele G., Mendoza R.G., Winnier D., Fiorentino T.V., Pengou Z., Cornell J., Andreozzi F., Jenkinson C., Cersosimo E., Federici M. (2014). The inflammatory status score including IL-6, TNF-α, osteopontin, fractalkine, MCP-1 and adiponectin underlies whole-body insulin resistance and hyperglycemia in type 2 diabetes mellitus. Acta Diabetol..

[16] Kadowaki T., Yamauchi T., Kubota N., Hara K., Ueki K., Tobe K. (2006). Adiponectin and adiponectin receptors in insulin re-sistance, diabetes, and the metabolic syndrome. J. Clin. Investig..

[17] Yu J.G., Javorschi S., Hevener A.L., Kruszynska Y.T., Norman R.A., Sinha M., Olefsky J.M. (2002). The effect of thiazolidinedi-ones on plasma adiponectin levels in normal, obese, and type diabetic subjects. Diabetes.

[18] Fonseca V. (2003). Effect of thiazolidinediones on body weight in patients with diabetes mellitus. Am. J. Med..

[19] Lago R.M., Singh P.P., Nesto R.W. (2007). Congestive heart failure and cardiovascular death in patients with prediabetes and type 2 diabetes given thiazolidinediones: A meta-analysis of randomized clinic trials. Lancet.

[20] Wang L., Waltenberger B., Pferschy-Wenzig E.-M., Blunder M., Liu X., Malainer C., Blazevic T., Schwaiger S., Rollinger J.M., Heiss E. (2014). Natural product agonists of peroxisome proliferator-activated receptor gamma (PPARγ): A review. Biochem. Pharmacol..

[21] Nakashima K., Murakami T., Tanabe H., Inoue M. (2014). Identification of a naturally occurring retinoid X receptor agonist from Brazilian green propolis. Biochim. Biophys. Acta.

[22] Wise L., Green H. (1979). Participation of one isozyme of cytosolic glycerophosphate dehydrogenase in the adipose conversion of 3T3 cells. J. Biol. Chem..

[23] Bankova V., Marcucci M.C., Simova S., Nikolova N., Kujumgiev A., Popov S. (1996). Anti-bacterial diterpenic acids from Bra-zilian propolis. Z. Nat. C.

[24] Sforcin J.M., Bankova V. (2011). Propolis: Is there a potential for the development of new drugs?. J. Ethnopharmacol..

[25] Prabhakar P.K., Doble M. (2011). Interaction of Cinnamic Acid Derivatives with Commercial Hypoglycemic Drugs on 2-Deoxyglucose Uptake in 3T3-L1 Adipocytes. J. Agric. Food Chem..

[26] Koya-Miyata S., Koya-Miyata S., Arai N., Mizote A., Taniguchi Y., Ushio S., Iwaki K., Fukuda S. (2009). Propolis prevents di-et-induced hyperlipidemia and mitigates weight gain in diet-induced obesity in mice. Biol. Pharm. Bull..

[27] Sakai T., Ohhata M., Fujii M., Oda S., Kusaka Y., Matsumoto M., Nakamoto A., Taki T., Nakamoto M., Shuto E. (2017). Brazilian Green Propolis Promotes Weight Loss and Reduces Fat Accumulation in C57BL/6 Mice Fed A High-Fat Diet. Biol. Pharm. Bull..

[28] Kakuta H., Kurosaki E., Niimi T., Gato K., Kawasaki Y., Suwa A., Honbou K., Yamaguchi T., Okumura H., Sanagi M. (2014). Distinct Properties of Telmisartan on Agonistic Activities for Peroxisome Proliferator-Activated Receptor γ among Clinically Used Angiotensin II Receptor Blockers: Drug-Target Interaction Analyses. J. Pharmacol. Exp. Ther..

[29] Schupp M., Clemenz M., Gineste R., Witt H., Janke J., Helleboid S., Hennuyer N., Ruiz P., Unger T., Staels B. (2005). Molecular characterization of new selective peroxisome proliferator-activated receptor gamma modula-tors with angiotensin receptor blocking activity. Diabetes.

[30] Aga H., Shibuya T., Sugimoto T., Kurimoto M., Nakajima S. (1994). Isolation and Identification of Antimicrobial Compounds in Brazilian Propolis. Biosci. Biotechnol. Biochem..

[31] Hattori H., Okuda K., Murase T., Shigetsura Y., Narise K., Semenza G.L., Nagasawa H. (2011). Isolation, identification, and biological evaluation of HIF-1-modulating compounds from Brazilian green propolis. Bioorg. Med. Chem..

